# Supplementary material for: Protocol for developing quality assurance measures to use in surgical trials: an example from the ROMIO study
Source: BMJ Open. 2019 Mar 1;9(3):e026209. doi: 10.1136/bmjopen-2018-026209 (PMC6429900; doi:10.1136/bmjopen-2018-026209)

BMJ Open

BMJ Open is committed to open peer review. As part of this commitment we make the peer review history of every article we publish publicly available.

When an article is published we post the peer reviewers' comments and the authors' responses online. We also post the versions of the paper that were used during peer review. These are the versions that the peer review comments apply to.

The versions of the paper that follow are the versions that were submitted during the peer review process. They are not the versions of record or the final published versions. They should not be cited or distributed as the published version of this manuscript.

BMJ Open is an open access journal and the full, final, typeset and author-corrected version of record of the manuscript is available on our site with no access controls, subscription charges or pay-per-view fees (http://bmjopen.bmj.com).

If you have any questions on BMJ Open's open peer review process please email info.bmjopen@bmj.com

BMJ Open

A protocol for developing quality assurance measures to use in pragmatic surgical trials: an example from the ROMIO study

Journal:	*BMJ Open*
Manuscript ID	bmjopen-2018-026209
Article Type:	Protocol
Date Submitted by the Author:	08-Sep-2018
Complete List of Authors:	Blencowe, Natalie; University of Bristol, Centre for Surgical Research, School of Social and Community Medicine<br>Skilton, Anni; University Hospitals Bristol NHS Foundation Trust, Medical Illustration<br>Gaunt, Daisy; University of Bristol Faculty of Medicine and Dentistry, Bristol Randomised Trials Collaboration & School of Social and Community Medicine; University of Bristol<br>Brierley, Rachel; University of Bristol<br>Hollowood, Andrew; University Hospitals Bristol NHS Foundation Trust<br>Dwerryhouse, Simon; Gloucestershire Hospitals NHS Foundation Trust<br>Higgs, Simon; Gloucestershire Hospitals NHS Foundation Trust<br>Robb, William; Beaumont Hospital<br>Boddy, Alex; University Hospitals of Leicester NHS Trust<br>Hanna, George; Imperial College, Faculty of Medicine, Department of Surgery & Cancer<br>Barham, C; University Hospitals Bristol NHS Foundation Trust, Division of Surgery<br>Blazeby, Jane; University of Bristol, Department of Social Medicine
Keywords:	randomised controlled trial, SURGERY, quality assurance

SCHOLARONE™
Manuscripts

A protocol for developing quality assurance measures to use in pragmatic surgical trials: an example from the ROMIO study

The ROMIO Study team

The ROMIO Study team is comprised of the following groups:

Writing group: Natalie S Blencowe[1,2*], Anni Skilton[1,3], Daisy Gaunt[1], Rachel Brierley[4], Andrew D Hollowood[2], Simon J Dwerryhouse[5], Simon M Higgs[5], William Robb[6], Alexander P Boddy[7], George B Hanna[8], C Paul Barham[2], Jane M Blazeby[1,2]

Affiliations:

1. Centre for Surgical Research, Population Health Sciences, Bristol Medical School, Bristol, UK

2. Division of Surgery, University Hospitals Bristol NHS Foundation Trust, Bristol, UK

3. Department of Medical Illustration, University Hospitals Bristol NHS Foundation Trust, Bristol, UK

4. Clinical Trials and Evaluation Unit, University of Bristol, University Hospitals Bristol NHS Foundation Trust, Bristol, UK

5. Department of Upper GI Surgery, Gloucestershire NHS Foundation Trust, Gloucester, UK

6. Department of Upper GI Surgery, Beaumont Hospital, Beaumont Road, Dublin, Ireland

7. Department of Upper GI Surgery, Leicester Royal Infirmary, Leicester, UK

8. Faculty of Medicine, Department of Surgery and Cancer, Imperial College London, UK

**Corresponding author:* natalie.blencowe@bristol.ac.uk

Co-applicants: Chris Metcalfe, Jenny Donovan, Newton Wong, Richard Berrisford, Benjamin Howes, William Hollingworth, Chris Rogers, Kerry Avery, Jackie Elliott.

Other members of the ROMIO study: Lucy Culliford, Marcus Jepson, Peter Lamb, Ravinder Vohra, James Catton, Rachel Melhado, Kish Pursani, Richard Krysztopik, James Byrne, Bilal Alkhaffaf, Tim

For peer review only - http://bmjopen.bmj.com/site/about/guidelines.xhtml

Underwood, Paul Wilkerson, Christopher Streets, Dan Titcomb, Richard Berrisford, Lee Humphreys, Tim Wheatley, Grant Sanders, Arun Ariyarathenam, James Byrne, Jamie Kelly, Fergus Noble, Graeme Couper, Richard Skipworth, Chris Deans, Anna Paisley, Sukhir Ubhi, Rob Williams, David Bowrey, David Exon, Paul Turner, Vinutha Shetty, Ram Chaparala, Khurshid Akhtar, Siba Senapati, Simon Parsons, Neil Welch, Naheed Farooq

Word count: 2387

Keywords: Randomised controlled trial, surgery, quality assurance

Article Summary

Strengths and limitations of this study

- This protocol describes the process of developing QA measures to use within pragmatic surgical RCTs, which is an area lacking in methodological guidance
- The increasing availability and use of digital imaging and information technologies will facilitate the use of these methods
- Further work will establish if these QA approaches can be used more widely in different contexts

Current progress

Ten study centres have now opened and are recruiting patients. 58 videos and 154 sets of intra-operative photos have been uploaded onto the study database.

For peer review only - http://bmjopen.bmj.com/site/about/guidelines.xhtml

Abstract

Introduction: Randomised controlled trials (RCTs) in surgery are frequently criticised because surgeon expertise and standards of surgery are not considered or accounted for during study design. This is particularly true in pragmatic trials (which typically involve multiple centres and surgeons and are based in 'real world' settings), compared with explanatory trials (which are smaller and more tightly controlled).

Objective: This protocol describes a process to develop and test quality assurance (QA) measures for use within a pragmatic surgical RCT comparing minimally invasive and open techniques for oesophageal cancer (the NIHR ROMIO study). It builds on methods initiated in the ROMIO pilot RCT. *Methods and analysis:* We have identified three distinct types of QA measure: i) entry criteria for surgeons, through assessment of operative videos, ii) standardization of operative techniques (by establishing minimum key procedural phases), and iii) monitoring of surgeons during the trial, using intra-operative photography to document key procedural phases and standardizing the pathological assessment of specimens. The QA measures will be adapted from the pilot study and tested iteratively, and the video and photo assessment tools will be tested for reliability and validity.

Ethics and dissemination: Ethics approval was obtained (NRES Committee South West - Frenchay, 25/04/2016, ref: 16/SW/0098). Results of the QA development study will be submitted for publication in a peer-reviewed journal.

Trial registration number: Pilot study ISRCTN59036820, main study ISRCTN10386621

For peer review only

For peer review only - http://bmjopen.bmj.com/site/about/guidelines.xhtml

Introduction and rationale

Randomized controlled trials (RCTs) in surgery are notoriously difficult to design and conduct, due to numerous methodological and cultural challenges. Many of these challenges relate to the fact that surgical procedures are complex healthcare interventions, meaning that 'unlike 20 milligram tablets, no two procedures are the same' and achieving standardization of surgical techniques and processes is difficult[1]. This is partly because surgeons naturally undertake procedures in (slightly) different ways and have differing skill levels. There is emerging empirical evidence that surgeons' technical skill may influence rates of post-operative complications and re-operation[2], and in the context of RCTs this may introduce bias. It is, therefore, important to provide reassurance about the standards of surgery in an RCT. This may be particularly pertinent in pragmatic trials, which are based in 'real world' settings and typically involve multiple centres and surgeons[3]. One way of achieving this is to undertake quality assurance, defined as the process(es) of 'directing the performance and behaviours of practitioners and institutions toward more appropriate and acceptable health outcomes'[4].

Undertaking quality assurance (QA) in surgery has been summarised in a systematic review of laparoscopic colorectal surgical studies[5]. The review identified three distinct categories of QA measures: i) trial entry criteria for surgeons and centres, ii) standardization of surgical techniques, and iii) monitoring of surgeons and/or units. Despite this, the use of such QA measures is rarely reported. In addition, it did not consider how QA processes may differ between pragmatic and explanatory trials. A recent systematic review of 80 RCTs found that 18% used entry criteria for surgeons or centres, 29% attempted to standardize the surgical procedures under evaluation (although most did not describe what the standards were), and 28% undertook some form of monitoring during the trial[6]. An additional problem is that the QA processes were often selected arbitrarily and relied on surgeons' self-reported data, rather than objective measurements, which leaves them open to criticism. Practical, robust approaches to quality assurance (QA) in pragmatic

For peer review only - http://bmjopen.bmj.com/site/about/guidelines.xhtml

surgical studies are lacking. The aim of this study, therefore, was to develop QA processes for pragmatic surgical trials, in the context of an RCT evaluating surgical techniques in upper GI cancer surgery (ROMIO: Randomised Oesophagectomy – Minimally Invasive or Open, HTA 14/140/78)[7].

The ROMIO study

The purpose of the ROMIO study is to compare, in patients with cancer of the oesophagus and oesophago-gastric junction, the clinical and cost-effectiveness of laparoscopically assisted (LAO) and open (OO) surgical procedures in terms of recovery, health related quality of life, cost and survival. The RCT will be conducted in at least eight UK centres. Assessing quality assurance (QA) is crucial, to ensure that the laparoscopically-assisted procedure (LAO) is performed to a similar standard as the open operation (OO), enabling a fair comparison to be made between the two techniques. Within the ROMIO pilot study, work was undertaken to begin the process of establishing QA methods, although the different requirements of pragmatic or explanatory studies was not considered[8]. This protocol outlines plans to test and modify these QA methods for the purposes of a multicentre, pragmatic study, the findings from which are required to be as generalisable as possible.

Objectives

- To examine the variability of performance of oesophagectomy, and agree on standardisation of surgery that are acceptable for pragmatic multicentre trial
- To pilot a tool to assess the quality of oesophagectomy undertaken within the ROMIO study
- To pilot a tool to enable ongoing monitoring of surgeons' technical performance throughout the RCT
- To explore the feasibility of using intra-operative digital photography and videos as methods for assessing QA in an RCT

For peer review only - http://bmjopen.bmj.com/site/about/guidelines.xhtml

- To develop a feedback system for surgeons participating in the trial

Methods and analysis

Methods to assess QA will be developed from work initiated in the pilot RCT. There are three categories: i) entry criteria for surgeons and centres, ii) standardization of surgical techniques, and iii) monitoring of surgeons and centres during the trial.

For peer review only

Entry criteria for centres

The criteria used to select centres to participate in the ROMIO study will be based on discussions within the study management group, informed by: recommendations for cancer centres (>50 cases per year), experience of team working in trials (agreement of at least two surgeons to enter patients into the trial), and commitment (shown by the provision of centre-level data for submission to the National Oesophago-Gastric Audit).

Entry criteria for surgeons

The feasibility of collecting videos of the abdominal phase of OO will be established. If found to be possible, participating surgeons will be required to submit one unedited video of the abdominal phase of OO (the 'standard' technique), in line with existing literature[2]. Because LAO represents a 'new' technique, and to prevent surgeons from selecting only their 'best' example, two unedited videos of the abdominal phase of LAO will be required. The videos will be pseudonymised and used to assess surgeons' overall technical skills, compare the quality of LAO and OO, understand and describe the variation in practice, and inform the debate around standards of surgery in the trial.

For peer review only - http://bmjopen.bmj.com/site/about/guidelines.xhtml

A schema outlining the proposed development and validation of the video assessment tool is provided in Figure 1. Current available methods for assessing the quality of surgeons' technical skills from videos include hierarchical task analysis[8] and the Objective Structured Assessment of Technical Skills (OSATS) tool[9]. OSATS is suitable for use with any type of surgical procedure (although has not been formally tested in the context of oesophagectomy) whereas hierarchical task analyses are developed individually for specific procedures. A hierarchical task analysis for oesophagectomy (HTA-O) was developed during the pilot phase of the ROMIO study[8]; however, it has not yet been formally tested. Both OSATS and HTA-O measures will be piloted simultaneously using 'think aloud' techniques, whereby a researcher will observe a surgeon (from the review team, see 'data analysis' section below) whilst reviewing an operative video[10]. The surgeon will be asked to complete each measure and express their thoughts whilst doing so, and to vocalise any general feelings about the technical skills displayed on the video. The 'think aloud' sessions will be audio recorded, transcribed verbatim and analysed thematically. Based on the findings from this process, the existing measures (OSATS and HTA-O) may be amended or combined, and new domains added. The 'think aloud' process will be iterative and stop once the study team are satisfied that no new amendments are necessary. It is anticipated that two surgeons will be involved in this piloting phase.

Standardization of surgical techniques

This phase of QA will be undertaken with careful consideration of the need to balance extensive standardization with the pragmatic nature of the study, and the practical challenges associated with monitoring adherence to the standards. LAO and OO will be deconstructed into their component parts, the 'key operative components' identified, and the expected similarities and differences for each component of both procedures will be mapped and documented using a typology of surgical interventions[11]. Details of how each component is recommended to be performed (and the degree

For peer review only - http://bmjopen.bmj.com/site/about/guidelines.xhtml

of flexibility permitted) will be agreed based on evidence from existing literature and consensus amongst the study team.

Monitoring of surgery during the trial

A schema outlining the piloting of a 'photo metric' of intra-operative quality assurance for oesophagectomy is provided in Figure 2. The photo metric is based on the premise that identification of specific anatomical structures on a photograph can be used as a measure of the quality of operative technique (because if the quality is poor, the structures would not be clearly visible)[12]. A list of anatomical structures that would be expected to be visible during each key component (as identified above) of LAO and OO was compiled during the pilot phase of the ROMIO study[8]. This provisional list will be refined by pilot testing in the operating theatre, to establish whether it is technically and logistically possible to collect photos of sufficient quality to demonstrate the required anatomical structures. The refined list will be discussed and agreed by surgeons participating in the ROMIO study. Subsequently, a rating scale will be developed (to include a category to represent that the anatomy cannot be seen or assessed) and the 'photo metric' will undergo reliability and validity testing.

All trial pathology specimens will be prepared and macroscopically and microscopically assessed in a uniform manner. The pathology data for the trial will be collected using a standardised form and represent data points included within the Royal College of Pathologists Dataset. Data points that will serve as surgical quality assurance indicators include the length of the oesophagus and the number of harvested lymph nodes. The slides of 10% of all cases from each centre will be reviewed by the Lead Pathologist. Pathologists will be blinded to the randomised allocation for each sample.

Data collection

For peer review only - http://bmjopen.bmj.com/site/about/guidelines.xhtml

Patients will be asked to give written informed consent for video and photographic recordings of the procedure, and transfer of the data to the trial co-ordinating centre (using local and study consent forms). LAO videos will be collected directly from the laparoscopy 'stack' already in routine use for the operations. Video recording will start from when the surgeon inserts the camera port and will end when the camera is removed after the procedure. Processes for obtaining videos of OO will be established as part of this study. Photographs will be taken using the laparoscopy ‘stack’ (for LAO and OO) and collected for each of the key phases identified during the ‘standardization of surgical procedures’ process (described above).

Feasibility data

An additional objective of this study is to collect information about the feasibility of developing QA processes involving the collection of digital images. Technical issues (and their solutions, if appropriate) will be documented as they arise and ‘standard operating procedure’ documents will be produced for both the video and photography QA aspects of this study.

Contextual information

We will record the total number of procedures performed by each participating surgeon, annual procedural volume and the total volume of oesophagectomies performed in each centre. The number of trainees undertaking procedures under supervision, and the number of procedures involving trainees, will be documented.

Data analysis

Rating of videos and photos will be undertaken by a team of oesophago-gastric surgeons: two participating in the ROMIO study and three routinely undertaking oesophagectomy outside of the

For peer review only - http://bmjopen.bmj.com/site/about/guidelines.xhtml

trial. Each video and photo will be rated by at least two surgeons. As with similar previous studies[2], no formal training or guidance will be given regarding the assessment tools. The 'rater' surgeons will not be aware of the surgeon or centre from which the videos and photos were obtained.

Video assessments (surgeon entry criteria)

Where videos are incomplete (e.g. a component of the operation has not been captured), the surgeon will be asked to provide a further video. Videos of surgeons receiving a summary judgement of 'poor' skill (or worse) will automatically be discussed and reviewed by the study team.

Photo assessments (monitoring)

The scoring system will be used to establish a threshold at which the standard of surgery is considered 'sufficient'. This will be determined by iterative review of all photos in discussion with members of the study management group. All photos considered not to meet this standard will be reviewed by the study team. Photo assessments will be correlated with the corresponding lymph node yield for each patient.

Feedback

Individual feedback

In cases where videos or photos do not meet the expected quality (in terms of the quality of the images as well as the standard of surgery), individualised and private feedback will be provided. In such instances, further operative videos may be requested to clarify that the feedback points have been addressed. However, given that the participants are all consultant oesophago-gastric surgeons, we anticipate that this will rarely occur.

Group feedback

For peer review only - http://bmjopen.bmj.com/site/about/guidelines.xhtml

To improve the overall quality of operations, which is another important part of QA, we will develop generic feedback materials for participating surgeons and centres. The generic feedback materials will be developed in two phases: i) understanding operative techniques, variations and difficulties (by watching the videos and documenting emerging patterns and themes), and ii) developing an action plan to address the identified difficulties and optimise operative techniques. We will achieve the second phase by developing short videos demonstrating operative techniques, based on the themes detected across the surgeons' submitted videos. These videos will be sent to all participating surgeons at regular intervals during the study. The key issues will also be discussed at ROMIO study investigators' meetings where exemplar videos of 'excellence' and 'room for improvement' will be displayed to allow self-reflection and learning.

After participating in the ROMIO study for 12 months, we will ask surgeons to submit further operative videos, to review progress and standards.

Public and patient involvement

Patients and the public were extensively involved in the design of the ROMIO study. During these meetings we asked for their views about the quality assurance aspect. They felt it was an important aspect of the RCT and did not have any issues relating to the acquisition of video and photo data relating to their operation.

Ethics and dissemination

Ethical approval for the quality assurance work was obtained as part of the ROMIO study. Digital videos will be transferred using ODIE (Open Document Information Exchange) to the NHS network for analysis by the ROMIO study team and pseudonymised with a unique identifier. The ODIE file

For peer review only - http://bmjopen.bmj.com/site/about/guidelines.xhtml

hosting system is securely protected through use of an encrypted HTTPS link, meaning third parties cannot read exchanged data. Digital photographs will be uploaded directly by the local site staff into the purpose-designed server ROMIO database hosted on the NHS network. Information capable of identifying individuals will be held in the database with passwords restricted to ROMIO study staff.

Results of the study will be submitted for publication in a peer-reviewed journal and presented at national and international gastrointestinal conferences. Guidance documents relating to: i) practical considerations for the development of QA procedures in surgical RCTs, and ii) generation of feedback materials to improve QA, will also be published. Feedback materials (in the form of 'gold standard' intra-operative photos of each key phase, and operative technique videos) will also be made available.

For peer review only

Figure 1. Schema for the development of a pre-trial QA tool to assess surgeons' overall operative technique in relation to oesophagectomy

Figure 2. Schema for the development of a QA tool to document the adequacy of crucial intra-operative steps building on work done in the pilot RCT

For peer review only - http://bmjopen.bmj.com/site/about/guidelines.xhtml

Funding: The ROMIO study (Randomised Oesophagectomy – Minimally Invasive or Open) is funded by the NIHR HTA (HTA 14/140/78). The quality assurance aspect of the ROMIO study was supported by the MRC ConDuCT-II (Collaboration and innovation for Difficult and Complex randomised controlled Trials In Invasive procedures) Hub for Trials Methodology Research (MR/K025643/1), and the NIHR Biomedical Research Centre at the University Hospitals Bristol NHS Foundation Trust and the University of Bristol. The views expressed in this publication are those of the author(s) and not necessarily those of the NHS, the National Institute for Health Research or the Department of Health. JMB is an NIHR Senior Investigator and NSB is an NIHR Academic Clinical Lecturer.

Competing interests: None to declare

Author contributions: All authors contributed to the study design and approved the final manuscript. NB, JMB, AS, GBH and CPB developed the video and photo tools. ADH, SJD, SMH, WR, APB and CPB tested the video tools and have undertaken assessments. NB wrote the first draft of the manuscript.

Data sharing: No further data available at this time (this is a protocol paper).

For peer review only

For peer review only - http://bmjopen.bmj.com/site/about/guidelines.xhtml

References

1. Lilford R, Braunholtz D, Harris J, *et al*. Trials in surgery. *Br J Surg* 2004; 91: 6–16.

2. Birkmeyer JD, Finks JF, O'Reilly A *et al*. Surgical skill and complication rates after bariatric surgery. *NEJM* 2013; 369:1434-1442

3. Zwarenstein M. Treweek S, Gagnier JJ, Altman DG, Tunis S, Haynes B *et al.* Improving the reporting of pragmatic trials: an extension of the CONSORT statement. *BMJ* 2008; 337:a2390

4. Palmer RH, Donabedian A, Povar G. Striving for quality in health care; an inquiry into policy and practice, Health Administration Press, Ann Arbor (1991), pp. xi-xvi

5. Foster JD, Mackenzie H, Nelson H, Hanna GB, Francis N. Methods of quality assurance in multicentre trials in laparoscopic colorectal surgery: a systematic review. *Ann Surg* 2014; 260(2): 220-229

6. Blencowe NS, Boddy AP, Harris A, Hanna T, Whiting P, Cook JA, Blazeby JM. Systematic review of intervention design and delivery in pragmatic and explanatory surgical randomized clinical trials. *Br J Surg* 2015; 102(9): 1037-1047

7. Avery KN, Metcalfe C, Berrisford R *et al*. The feasibility of a randomized controlled trial of esophagectomy for esophageal cancer--the ROMIO (Randomized Oesophagectomy: Minimally Invasive or Open) study: protocol for a randomized controlled trial. *Trials* 2014; 15:200

8. Metcalfe C, Avery K, Berrisford R *et al*. Comparing open and minimally invasive surgical procedures for oesophagectomy in the treatment of cancer: the ROMIO (Randomised Oesophagectomy: Minimally Invasive or Open) feasibility study and pilot trial. *Health Technol Assess.* 2016; 20(48):1-68

9. Martin JA, Regehr G, Reznick R *et al.* Objective structured assessment of technical skill (OSATS) for surgical residents. *Br J Surg* 1997; 84(2):273-8

10. Charters E. The use of think-aloud methods in qualitative research: an introduction to think-aloud methods. *Brock Education* 2003; 12(2):68-82

11. Blencowe NS, Mills N, Cook JA, Donovan JL, Rogers CA, Whiting P, Blazeby JM. Standardizing and monitoring the delivery of surgical interventions in randomized clinical trials. *Br J Surg* 2016; 103(10); 1377-1384

12. Murphy BL, Boughey JC, Degnim AC *et al.* A picture is worth a thousand words: intraoperative photography as a quality metric for axillary dissection. *Ann Surg Oncol* 2016; 23:3494-3500

For peer review only - http://bmjopen.bmj.com/site/about/guidelines.xhtml

Figure 1. Schema for the development of a pre-trial QA tool to assess surgeons' overall operative technique in relation to oesophagectomy

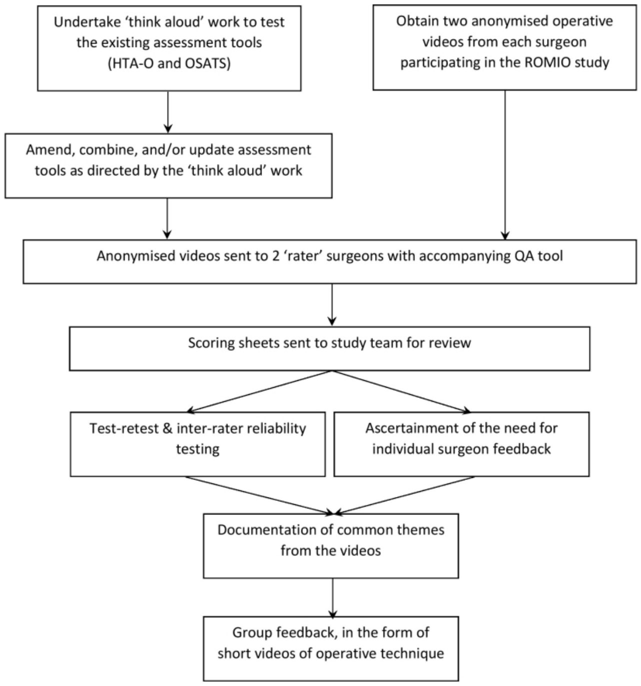

HTA-O = Hierarchical Task Analysis for Oesophagectomy[8]

OSATS = Objective Structured Assessment of Technical Skills[9]

209x297mm (300 x 300 DPI)

Figure 2. Schema for the development of a QA tool to document the adequacy of crucial intra-operative steps building on work done in the pilot RCT

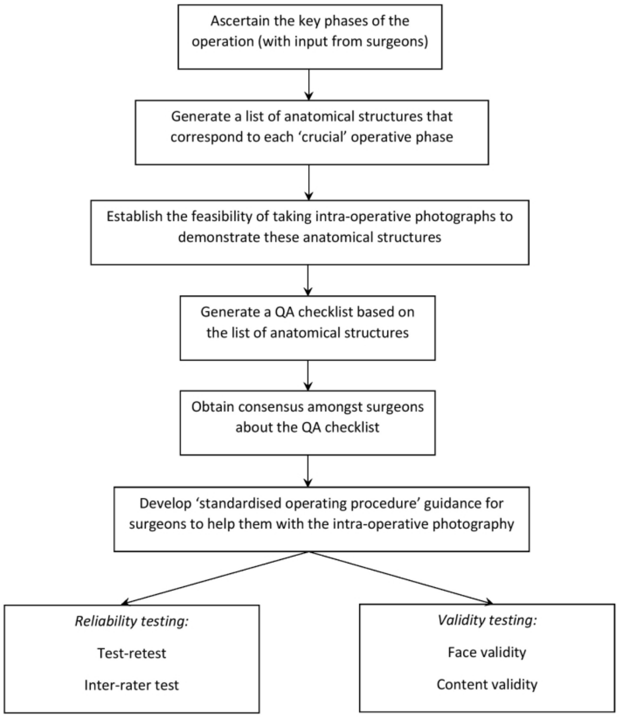

209x297mm (300 x 300 DPI)

BMJ Open

A protocol for developing quality assurance measures to use in surgical trials: an example from the ROMIO study

Journal:	*BMJ Open*
Manuscript ID	bmjopen-2018-026209.R1
Article Type:	Protocol
Date Submitted by the Author:	08-Dec-2018
Complete List of Authors:	Blencowe, Natalie; University of Bristol, Centre for Surgical Research, School of Social and Community Medicine<br>Skilton, Anni; University Hospitals Bristol NHS Foundation Trust, Medical Illustration<br>Gaunt, Daisy; University of Bristol Faculty of Medicine and Dentistry, Bristol Randomised Trials Collaboration & School of Social and Community Medicine; University of Bristol<br>Brierley, Rachel; University of Bristol<br>Hollowood, Andrew; University Hospitals Bristol NHS Foundation Trust<br>Dwerryhouse, Simon; Gloucestershire Hospitals NHS Foundation Trust<br>Higgs, Simon; Gloucestershire Hospitals NHS Foundation Trust<br>Robb, William; Beaumont Hospital<br>Boddy, Alex; University Hospitals of Leicester NHS Trust<br>Hanna, George; Imperial College, Faculty of Medicine, Department of Surgery & Cancer<br>Barham, C; University Hospitals Bristol NHS Foundation Trust, Division of Surgery<br>Blazeby, Jane; University of Bristol, Department of Social Medicine
**Primary Subject Heading**:	Surgery
Secondary Subject Heading:	Health services research
Keywords:	randomised controlled trial, SURGERY, quality assurance

SCHOLARONE™
Manuscripts

A protocol for developing quality assurance measures to use in surgical trials: an example from the ROMIO study

The ROMIO Study team

The ROMIO Study team is comprised of the following groups:

Writing group: Natalie S Blencowe[1,2*], Anni Skilton[1,3], Daisy Gaunt[1], Rachel Brierley[4], Andrew D Hollowood[2], Simon J Dwerryhouse[5], Simon M Higgs[5], William Robb[6], Alexander P Boddy[7], George B Hanna[8], C Paul Barham[2], Jane M Blazeby[1,2]

Affiliations:

1. Centre for Surgical Research, Population Health Sciences, Bristol Medical School, Bristol, UK

2. Division of Surgery, University Hospitals Bristol NHS Foundation Trust, Bristol, UK

3. Department of Medical Illustration, University Hospitals Bristol NHS Foundation Trust, Bristol, UK

4. Clinical Trials and Evaluation Unit, University of Bristol, University Hospitals Bristol NHS Foundation Trust, Bristol, UK

5. Department of Upper GI Surgery, Gloucestershire NHS Foundation Trust, Gloucester, UK

6. Department of Upper GI Surgery, Beaumont Hospital, Beaumont Road, Dublin, Ireland

7. Department of Upper GI Surgery, Leicester Royal Infirmary, Leicester, UK

8. Faculty of Medicine, Department of Surgery and Cancer, Imperial College London, UK

**Corresponding author:* natalie.blencowe@bristol.ac.uk

Co-applicants: Chris Metcalfe, Jenny Donovan, Newton Wong, Richard Berrisford, Benjamin Howes, William Hollingworth, Chris Rogers, Kerry Avery, Jackie Elliott.

Other members of the ROMIO study: Lucy Culliford, Marcus Jepson, Peter Lamb, Ravinder Vohra, James Catton, Rachel Melhado, Kish Pursani, Richard Krysztopik, James Byrne, Bilal Alkhaffaf, Tim Underwood, Paul Wilkerson, Christopher Streets, Dan Titcomb, Richard Berrisford, Lee Humphreys, Tim Wheatley, Grant Sanders, Arun Ariyarathenam, James Byrne, Jamie Kelly, Fergus Noble, Graeme

For peer review only

Couper, Richard Skipworth, Chris Deans, Anna Paisley, Sukhir Ubhi, Rob Williams, David Bowrey, David Exon, Paul Turner, Vinutha Shetty, Ram Chaparala, Khurshid Akhtar, Siba Senapati, Simon Parsons, Neil Welch, Naheed Farooq

Word count: 2387

Keywords: Randomised controlled trial, surgery, quality assurance

Current progress

Ten study centres have now opened and are recruiting patients. 58 videos and 154 sets of intra-operative photos have been uploaded onto the study database.

For peer review only

Abstract

Introduction: Randomised controlled trials (RCTs) in surgery are frequently criticised because surgeon expertise and standards of surgery are not considered or accounted for during study design. This is particularly true in pragmatic trials (which typically involve multiple centres and surgeons and are based in 'real world' settings), compared with explanatory trials (which are smaller and more tightly controlled).

Objective: This protocol describes a process to develop and test quality assurance (QA) measures for use within a predominantly pragmatic surgical RCT comparing minimally invasive and open techniques for oesophageal cancer (the NIHR ROMIO study). It builds on methods initiated in the ROMIO pilot RCT. *Methods and analysis:* We have identified three distinct types of QA measure: i) entry criteria for surgeons, through assessment of operative videos, ii) standardization of operative techniques (by establishing minimum key procedural phases), and iii) monitoring of surgeons during the trial, using intra-operative photography to document key procedural phases and standardizing the pathological

For peer review only - http://bmjopen.bmj.com/site/about/guidelines.xhtml

assessment of specimens. The QA measures will be adapted from the pilot study and tested iteratively, and the video and photo assessment tools will be tested for reliability and validity.

Ethics and dissemination: Ethics approval was obtained (NRES Committee South West - Frenchay, 25/04/2016, ref: 16/SW/0098). Results of the QA development study will be submitted for publication in a peer-reviewed journal.

Trial registration number: Pilot study ISRCTN59036820, main study ISRCTN10386621

Article Summary

Strengths and limitations of this study

- This protocol describes the process of developing QA measures to use within ~~pragmatic~~ surgical RCTs, which is an area lacking in methodological guidance
- Further work will establish if these QA approaches can be used more widely in different contexts
- This study will explore the feasibility of obtaining digital videos of open surgery during this study; however, it may not be possible to achieve this with good enough quality or without interfering with the process of surgery. The increasing availability and use of digital imaging and information technologies should facilitate the use of these methods
- QA processes require investment of resource and expertise: future work to streamline them is therefore needed
- Whilst the addition of QA processes can reduce bias and improve internal validity, they may initially appear to compromise a trial's generalisability. Provided pragmatic standards are set and monitored, this should be avoided

For peer review only

Introduction and rationale

Randomized controlled trials (RCTs) in surgery are notoriously difficult to design and conduct, due to numerous methodological and cultural challenges. Many of these challenges relate to the fact that surgical procedures are complex healthcare interventions, meaning that 'unlike 20 milligram tablets, no two procedures are the same' and achieving standardization of surgical techniques and processes is difficult[1]. This is partly because surgeons naturally undertake procedures in (slightly) different ways and have differing skill levels, which may influence rates of post-operative complications and re-operation[2]. A lack of consideration for intervention standardization and surgeon expertise in the context of RCTs may introduce bias, compromising internal validity. This is acknowledged in guidance such as CONSORT-NPT (an extension to the CONSORT statement for non-pharmacological treatments such as surgery) and SPIRIT, which provides a checklist of 33 items to be reported in trial protocols. They recommend reporting precise details of the intervention and its components to enable replication in routine practice, information about standardization, procedures for monitoring adherence to intervention protocols, and consideration for the expertise of care providers.

Neither CONSORT-NPT nor SPIRIT differentiate between the information required in pragmatic or explanatory settings. This may be because it is rare for trials to be purely pragmatic or explanatory: trial design is not dichotomous and there is a continuum between the two extremes. In explanatory trials, which determine the efficacy of interventions, great detail may be necessary because the interventions are often novel and their safety needs to be assessed within carefully controlled settings. Pragmatic trials, which determine whether interventions are effective in the real world, are often multicentre studies with large numbers of surgeons[3]. Under such circumstances, specifying each operative step is likely to create difficulties, and ensuring that each step was delivered as planned may be unrealistic. A balance between adequate standardization and practicality is therefore necessary and appropriate. One way of achieving this is to determine the minimum active ingredients of the intervention – those that are thought to optimize outcomes or those that are different between the

For peer review only - http://bmjopen.bmj.com/site/about/guidelines.xhtml

interventions in each trial group – and the degree to which they need to be standardized. In this way, monitoring only the key components may be sufficient, rather than monitoring all components and steps, in order to ensure the intervention is actually delivered as planned[4]. This approach would also account for the fact that most trials sit within the pragmatic-explanatory continuum rather than being one or the other.

It is, therefore, important to provide reassurance about the standards of surgery in all RCTs, whilst recognising that this may vary according to trial design. One way of achieving this is to undertake quality assurance, defined as the process(es) of 'directing the performance and behaviours of practitioners and institutions toward more appropriate and acceptable health outcomes'[5]. Undertaking quality assurance (QA) in surgery has been summarised in a systematic review of laparoscopic colorectal surgical studies[6]. The review identified three distinct categories of QA measures: i) trial entry criteria for surgeons and centres, ii) standardization of surgical techniques, and iii) monitoring of surgeons and/or units. Despite this, the use of such QA measures is rarely reported. In addition, it did not consider how QA processes may differ between pragmatic and explanatory trials. A recent systematic review of 80 RCTs found that 18% used entry criteria for surgeons or centres, 29% attempted to standardize the surgical procedures under evaluation (although most did not describe what the standards were), and 28% undertook some form of monitoring during the trial[7]. An additional problem is that the QA processes were often selected arbitrarily and relied on surgeons' self-reported data, rather than objective measurements, which leaves them open to criticism. Practical, robust approaches to quality assurance (QA) in ~~pragmatic~~ surgical studies are lacking. The aim of this study, therefore, was to develop and test QA processes for ~~pragmatic~~ surgical trials, in the context of a predominantly pragmatic RCT evaluating surgical techniques in upper GI cancer surgery (ROMIO: Randomised Oesophagectomy – Minimally Invasive or Open, HTA 14/140/78)[8,9].

The ROMIO study

For peer review only - http://bmjopen.bmj.com/site/about/guidelines.xhtml

The purpose of the ROMIO study is to compare, in patients with cancer of the oesophagus and oesophago-gastric junction, the clinical and cost-effectiveness of laparoscopically assisted (LAO) and open (OO) surgical procedures in terms of recovery, health related quality of life, cost and survival. The RCT will be conducted in at least eight UK centres. The ROMIO study is predominantly pragmatic, as demonstrated by the PRECIS-II (Pragmatic-Explanatory Continuum Indicator Summary) wheel provided in Figure 1[10]. For example, it is multi-centre and involves more than 40 surgeons. It has broad inclusion criteria and it is expected that at least 60% of patients undergoing oesophagectomy will be eligible to participate. The primary outcome is patient-centred and secondary outcomes include resource use and other clinical and patient reported outcomes. Despite this, however, assessing quality assurance (QA) is crucial, to ensure that the laparoscopically-assisted procedure (LAO) is performed to a similar standard as the open operation (OO), enabling a fair comparison to be made between the two techniques. Within the ROMIO pilot study, work was undertaken to begin the process of establishing QA methods. This protocol outlines plans to test and assess the feasibility of implementing these QA methods for the purposes of a multicentre RCT in surgery.

Objectives

- To examine the variability of performance of oesophagectomy, and agree on standardisation of surgery that are acceptable for a predominantly pragmatic multicentre trial
- To pilot a tool to assess the quality of oesophagectomy undertaken within the ROMIO study
- To pilot a tool to enable ongoing monitoring of surgeons' technical performance throughout the RCT
- To explore the feasibility of using intra-operative digital photography and videos as methods for assessing QA in an RCT
- To develop a feedback system for surgeons participating in the trial

For peer review only - http://bmjopen.bmj.com/site/about/guidelines.xhtml

Methods and analysis

Methods to assess QA will be developed from work initiated in the pilot RCT. There are three categories: i) entry criteria for surgeons and centres, ii) standardization of surgical techniques, and iii) monitoring of surgeons and centres during the trial. Methods to assess QA in each of these categories will carefully consider the balance between extensive selection (of centres and surgeons) and standardization (of technique) with the predominantly pragmatic nature of the study.

Entry criteria for centres

The criteria used to select centres to participate in the ROMIO study will be based on discussions within the study management group, informed by: recommendations for cancer centres (>50 cases per year), experience of team working in trials (agreement of at least two surgeons to enter patients into the trial), and commitment (shown by the provision of centre-level data for submission to the National Oesophago-Gastric Audit).

Entry criteria for surgeons

Rather than prevent surgeons from participating in the study, the purpose of this aspect of QA was predominantly to enable i) variations in surgical technique and skill to be described, facilitating contextualisation of the results, ii) provision of feedback, and iii) to establish whether LAO was broadly being performed to the same standard as OO. The feasibility of collecting videos of the abdominal phase of OO will be established. If found to be possible, participating surgeons will be required to submit one unedited video of the abdominal phase of OO (the 'standard' technique), in line with existing literature[2]. Because LAO represents a 'new' technique, and to prevent surgeons from selecting only their 'best' example, two unedited videos of the abdominal phase of LAO will be required. All videos will be pseudonymised.

For peer review only - http://bmjopen.bmj.com/site/about/guidelines.xhtml

A schema outlining the proposed development and validation of the video assessment tool is provided in Figure 2. Current available methods for assessing the quality of surgeons' technical skills from videos include hierarchical task analysis[9] and the Objective Structured Assessment of Technical Skills (OSATS) tool[11]. OSATS is suitable for use with any type of surgical procedure (although has not been formally tested in the context of oesophagectomy) whereas hierarchical task analyses are developed individually for specific procedures. A hierarchical task analysis for oesophagectomy (HTA-O) was developed during the pilot phase of the ROMIO study[9]; however, it has not yet been formally tested. Both OSATS and HTA-O measures will be piloted simultaneously using 'think aloud' techniques, whereby a researcher will observe a surgeon (from the review team, see 'data analysis' section below) whilst reviewing an operative video[12]. The surgeon will be asked to complete each measure and express their thoughts whilst doing so, and to vocalise any general feelings about the technical skills displayed on the video. The 'think aloud' sessions will be audio recorded, transcribed verbatim and analysed thematically. Based on the findings from this process, the existing measures (OSATS and HTA-O) may be amended or combined, and new domains added. The 'think aloud' process will be iterative – i.e. it will be repeated with different videos until the surgeon has no further comments - and stop once the study team are satisfied that no new amendments are necessary. It is anticipated that two surgeons will be involved in this piloting phase.

Standardization of surgical techniques

This phase of QA will be undertaken with careful consideration of the need to balance extensive standardization with the pragmatic nature of the study, and the practical challenges associated with monitoring adherence to the standards. LAO and OO will be deconstructed into their component parts, the 'key operative components' identified, and the expected similarities and differences for each component of both procedures will be mapped and documented using a typology of surgical interventions[13]. Details of how each component is recommended to be performed (and the degree of

For peer review only - http://bmjopen.bmj.com/site/about/guidelines.xhtml

flexibility permitted) will be agreed based on evidence from existing literature and consensus amongst the study team.

Monitoring of surgery during the trial

A schema outlining the piloting of a 'photo metric' of intra-operative quality assurance for oesophagectomy is provided in Figure 3. The photo metric is based on the premise that identification of specific anatomical structures on a photograph can be used as a measure of the quality of operative technique (because if the quality is poor, the structures would not be clearly visible)[14]. A list of anatomical structures that would be expected to be visible during each key component (as identified above) of LAO and OO was compiled during the pilot phase of the ROMIO study[9]. This provisional list will be refined by pilot testing in the operating theatre (out with the ROMIO study), to establish whether it is technically and logistically possible to collect photos of sufficient quality to demonstrate the required anatomical structures. The refined list will be discussed and agreed by surgeons participating in the ROMIO study. Subsequently, a rating scale will be developed (to include a category to represent that the anatomy cannot be seen or assessed) and the 'photo metric' will undergo reliability and validity testing. Surgeons will be encouraged to submit photos for each patient recruited to the ROMIO study, to demonstrate that the key operative components have been performed.

All trial pathology specimens will be prepared and macroscopically and microscopically assessed in a uniform manner. The pathology data for the trial will be collected using a standardised form and represent data points included within the Royal College of Pathologists Dataset. Data points that will serve as surgical quality assurance indicators include the length of the oesophagus and the number of harvested lymph nodes. The slides of 10% of all cases from each centre will be reviewed by the Lead Pathologist. Pathologists will be blinded to the randomised allocation for each sample.

For peer review only

Data collection

Patients will be asked to give written informed consent for video and photographic recordings of the procedure (separate to consent for recruitment into the ROMIO study itself), and transfer of the data to the trial co-ordinating centre (using local and study consent forms). LAO videos will be collected directly from the laparoscopy 'stack' already in routine use for the operations. Video recording will start from when the surgeon inserts the camera port and will end when the camera is removed after the procedure. Processes for obtaining videos of OO will be established as part of this study. Photographs will be taken using the laparoscopy 'stack' (for LAO and OO) and collected for each of the key phases identified during the 'standardization of surgical procedures' process (described above).

Feasibility data

An additional objective of this study is to collect information about the feasibility of developing QA processes involving the collection of digital images. Technical issues (and their solutions, if appropriate) will be documented as they arise and 'standard operating procedure' documents will be produced for both the video and photography QA aspects of this study. We envisage that it may be particularly problematic to collect digital videos of open surgery, that sufficiently match the quality of laparoscopic procedures. Specific attention will be paid to developing solutions to overcome the barriers to collecting such data, including the need to minimise any interference with the usual process of surgery.

Contextual information

We will record the total number of procedures performed by each participating surgeon, annual procedural volume and the total volume of oesophagectomies performed in each centre. The number

For peer review only

For peer review only - http://bmjopen.bmj.com/site/about/guidelines.xhtml

of trainees undertaking procedures under supervision, and the number of procedures involving trainees, will be documented.

Data analysis

Rating of videos and photos will be undertaken by a team of oesophago-gastric surgeons: two participating in the ROMIO study and three routinely undertaking oesophagectomy outside of the trial. Each video and photo will be rated by at least two surgeons. As with similar previous studies[2], no formal training or guidance will be given regarding the assessment tools. The 'rater' surgeons will not be aware of the surgeon or centre from which the videos and photos were obtained.

Video assessments (surgeon entry criteria)

Where videos are incomplete (e.g. a component of the operation has not been captured), the surgeon will be asked to provide a further video. Videos of surgeons receiving a summary judgement of 'poor' skill (or worse) will automatically be discussed and reviewed by the study team.

Photo assessments (monitoring)

The scoring system will be used to establish a threshold at which the standard of surgery is considered 'sufficient'. This will be determined by iterative review of all photos in discussion with members of the study management group. All photos considered not to meet this standard will be reviewed by the study team. Photo assessments will be correlated with the corresponding lymph node yield for each patient.

Feedback

Individual feedback

For peer review only - http://bmjopen.bmj.com/site/about/guidelines.xhtml

In cases where videos or photos do not meet the expected quality (in terms of the quality of the images as well as the standard of surgery), individualised and private feedback will be provided. In such instances, further operative videos may be requested to clarify that the feedback points have been addressed. However, given that the participants are all consultant oesophago-gastric surgeons, we anticipate that this will rarely occur.

Group feedback

To improve the overall quality of operations, which is another important part of QA, we will develop generic feedback materials for participating surgeons and centres. The generic feedback materials will be developed in two phases: i) understanding operative techniques, variations and difficulties (by watching the videos and documenting emerging patterns and themes), and ii) developing an action plan to address the identified difficulties and optimise operative techniques. We will achieve the second phase by developing short videos demonstrating operative techniques, based on the themes detected across the surgeons' submitted videos. These videos will be sent to all participating surgeons at regular intervals during the study. The key issues will also be discussed at ROMIO study investigators' meetings where exemplar videos of 'excellence' and 'room for improvement' will be displayed to allow self-reflection and learning.

After participating in the ROMIO study for 12 months, we will ask surgeons to submit further operative videos, to review progress and standards.

Future work

This study aims to develop methods for measuring the QA of surgical interventions. Once developed, they will be implemented in the context of an RCT comparing open and laparoscopic surgery for patients with oesophageal cancer. There are numerous analyses that may be undertaken, which will depend on the exact nature of the QA measures that are developed. First, the quality of intervention

For peer review only - http://bmjopen.bmj.com/site/about/guidelines.xhtml

delivery will be assessed, and to compare what surgeons reported in the case report forms (i.e. what they said they did) with the intra-operative photographs (i.e. what actually happened). Second, we will explore trends relating to surgeon skill (from the operative videos) and patient outcomes, though numbers are relatively small and this may not, therefore, be possible. Third, the process of developing QA measures for this study will influence future work in this area; specifically, the generation of guidance that can be extrapolated to other RCTs in surgery. This is the focus of a funded fellowship award, which will also examine the process of obtaining consensus about exactly what these QA measures should comprise, accounting for trial design and the nature of the interventions under investigation. Finally, it is important to recognise that improving the QA of surgical interventions may improve standards of surgery within an RCT. Whilst we will document the points at which feedback is given to surgeons and centres, it may not be realistically possible to correlate this (and assign causation) with outcomes. It is well recognised that patients within RCTs generally have more favourable outcomes than those that do not. Improvements to the standards of intervention delivery may, therefore, form a part of the benefits associated with trial participation.

Public and patient involvement

Patients and the public were extensively involved in the design of the ROMIO study. During these meetings we asked for their views about the quality assurance aspect. They felt it was an important aspect of the RCT and did not have any issues relating to the acquisition of video and photo data relating to their operation.

Ethics and dissemination

Ethical approval for the quality assurance work was obtained as part of the ROMIO study. Digital videos will be transferred using ODIE (Open Document Information Exchange) to the NHS network for

For peer review only - http://bmjopen.bmj.com/site/about/guidelines.xhtml

For peer review only

analysis by the ROMIO study team and pseudonymised with a unique identifier. The ODIE file hosting system is securely protected through use of an encrypted HTTPS link, meaning third parties cannot read exchanged data. Digital photographs will be uploaded directly by the local site staff into the purpose-designed server ROMIO database hosted on the NHS network. Information capable of identifying individuals will be held in the database with passwords restricted to ROMIO study staff.

Results of the study will be submitted for publication in a peer-reviewed journal and presented at national and international gastrointestinal conferences. Guidance documents relating to: i) practical considerations for the development of QA procedures in surgical RCTs, and ii) generation of feedback materials to improve QA, will also be published. Feedback materials (in the form of 'gold standard' intra-operative photos of each key phase, and operative technique videos) will also be made available.

Figure 1. PRECIS-II wheel: a visual representation of the predominantly pragmatic nature of the ROMIO study[10]

Figure 2. Schema for the development of a pre-trial QA tool to assess surgeons' overall operative technique in relation to oesophagectomy

Figure 3. Schema for the development of a QA tool (photo metric) to document the adequacy of crucial intra-operative steps building on work done in the pilot RCT

Funding: The ROMIO study (Randomised Oesophagectomy – Minimally Invasive or Open) is funded by the NIHR HTA (HTA 14/140/78). The quality assurance aspect of the ROMIO study was supported by the MRC ConDuCT-II (Collaboration and innovation for Difficult and Complex randomised controlled Trials In Invasive procedures) Hub for Trials Methodology Research (MR/K025643/1), and the NIHR Biomedical Research Centre at the University Hospitals Bristol NHS Foundation Trust and

For peer review only - http://bmjopen.bmj.com/site/about/guidelines.xhtml

the University of Bristol. The views expressed in this publication are those of the author(s) and not necessarily those of the NHS, the National Institute for Health Research or the Department of Health. JMB is an NIHR Senior Investigator and NSB is an NIHR Academic Clinical Lecturer.

Competing interests: None to declare

Author contributions: All authors contributed to the study design and approved the final manuscript. NB, JMB, AS, DG, RB, GBH and CPB developed the video and photo tools. ADH, SJD, SMH, WR, APB and CPB tested the video tools and have undertaken assessments. NB and JMB wrote the first draft of the manuscript.

Data sharing: No further data available at this time (this is a protocol paper).

For peer review only

For peer review only - http://bmjopen.bmj.com/site/about/guidelines.xhtml

References

1. Lilford R, Braunholtz D, Harris J, *et al*. Trials in surgery. *Br J Surg* 2004; 91: 6–16.

2. Birkmeyer JD, Finks JF, O'Reilly A *et al*. Surgical skill and complication rates after bariatric surgery. *NEJM* 2013; 369:1434-1442

3. Zwarenstein M. Treweek S, Gagnier JJ, Altman DG, Tunis S, Haynes B *et al.* Improving the reporting of pragmatic trials: an extension of the CONSORT statement. *BMJ* 2008; 337:a2390

4. Blencowe NS, Blazeby JM, Donovan JL, Mills N. Novel ways to explore surgical interventions in randomised controlled trials: applying case study methodology in the operating theatre. *Trials*2015; **16**: 589.

5. Palmer RH, Donabedian A, Povar G. Striving for quality in health care; an inquiry into policy and practice, Health Administration Press, Ann Arbor (1991), pp. xi-xvi

6. Foster JD, Mackenzie H, Nelson H, Hanna GB, Francis N. Methods of quality assurance in multicentre trials in laparoscopic colorectal surgery: a systematic review. *Ann Surg* 2014; 260(2): 220-229

7. Blencowe NS, Boddy AP, Harris A, Hanna T, Whiting P, Cook JA, Blazeby JM. Systematic review of intervention design and delivery in pragmatic and explanatory surgical randomized clinical trials. *Br J Surg* 2015; 102(9): 1037-1047

8. Avery KN, Metcalfe C, Berrisford R *et al*. The feasibility of a randomized controlled trial of esophagectomy for esophageal cancer--the ROMIO (Randomized Oesophagectomy: Minimally Invasive or Open) study: protocol for a randomized controlled trial. *Trials* 2014; 15:200

9. Metcalfe C, Avery K, Berrisford R *et al*. Comparing open and minimally invasive surgical procedures for oesophagectomy in the treatment of cancer: the ROMIO (Randomised Oesophagectomy: Minimally Invasive or Open) feasibility study and pilot trial. *Health Technol Assess.* 2016; 20(48):1-68

10. Loudon K, Treweek S, Sullivan F *et al*. The PRECIS 2 tool: designing trials that are fit for purpose. *BMJ* 2015;350:h2147

11. Martin JA, Regehr G, Reznick R *et al.* Objective structured assessment of technical skill (OSATS) for surgical residents. *Br J Surg* 1997; 84(2):273-8

12. Charters E. The use of think-aloud methods in qualitative research: an introduction to think-aloud methods. *Brock Education* 2003; 12(2):68-82

13. Blencowe NS, Mills N, Cook JA, Donovan JL, Rogers CA, Whiting P, Blazeby JM. Standardizing and monitoring the delivery of surgical interventions in randomized clinical trials. *Br J Surg* 2016; 103(10); 1377-1384

14. Murphy BL, Boughey JC, Degnim AC *et al.* A picture is worth a thousand words: intraoperative photography as a quality metric for axillary dissection. *Ann Surg Oncol* 2016; 23:3494-3500

For peer review only - http://bmjopen.bmj.com/site/about/guidelines.xhtml

Figure 1. PRECIS-II wheel: a visual representation of the predominantly pragmatic nature of the ROMIO study[10]

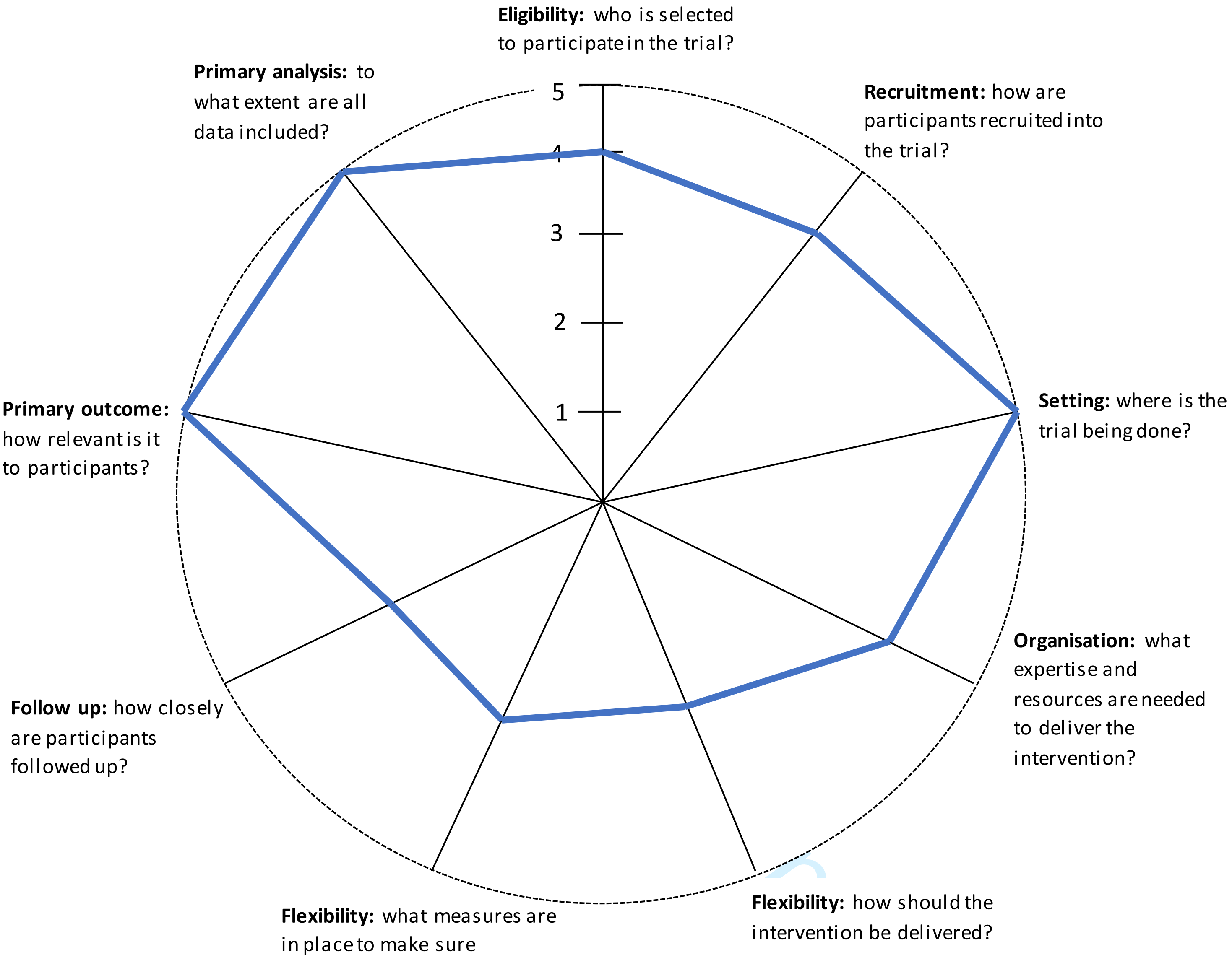

Trials that take an explanatory approach produce wheels nearer the hub; those with a pragmatic approach are closer to the rim.

Figure 2. Schema for the development of a pre-trial QA tool to assess surgeons' overall operative technique in relation to oesophagectomy

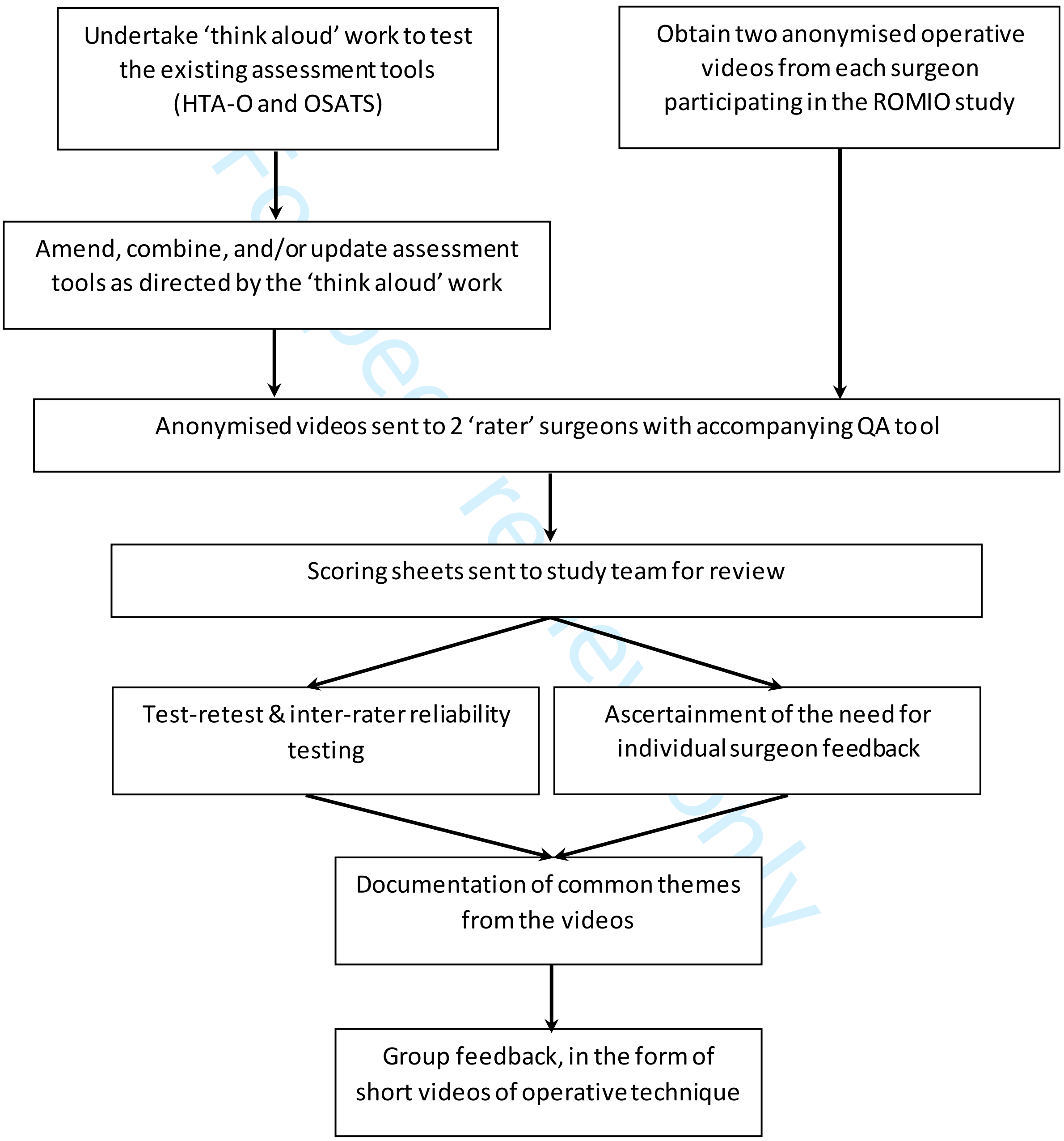

HTA-O = Hierarchical Task Analysis for Oesophagectomy[8]

OSATS = Objective Structured Assessment of Technical Skills[9]

Figure 3. Schema for the development of a QA tool (photo metric) to document the adequacy of crucial intra-operative steps building on work done in the pilot RCT

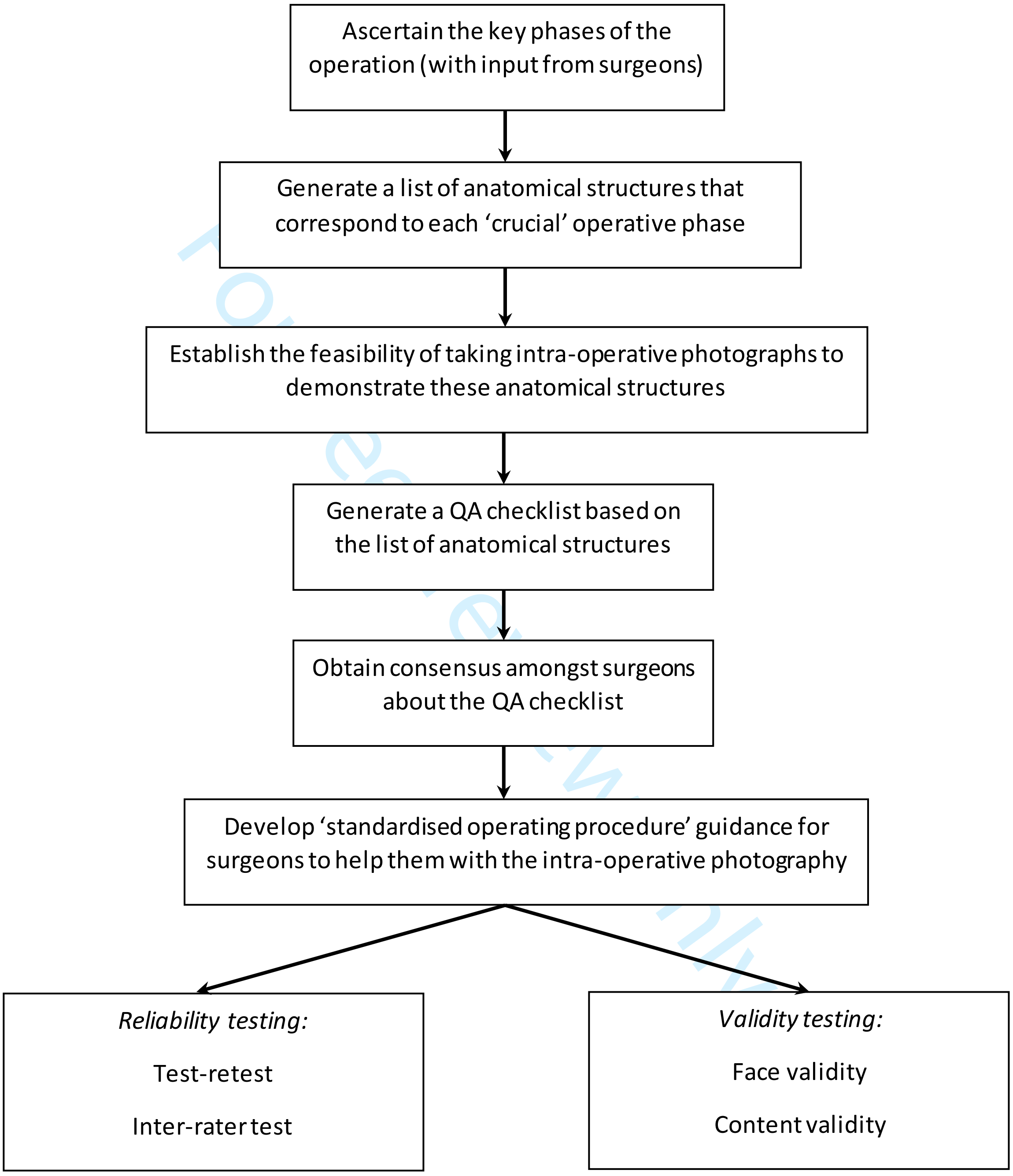

Supplement: Author's manuscript [file bmjopen-2018-026209.draft_revisions.pdf]
